# Non-traditional Surgical Treatment of a Rolando Fracture

**DOI:** 10.7759/cureus.69864

**Published:** 2024-09-21

**Authors:** Jakub Florek, Filip Georgiew, Oles Petrovych, Pawel Florek, Sebastian Janowiec

**Affiliations:** 1 Department of Orthopedics and Traumatology, Rydygier Hospital, Brzesko, POL; 2 Faculty of Health Science, University of Applied Science, Tarnow, POL

**Keywords:** arthroplasty, carpometacarpal, cmc, endoprosthesis, osteoarthritis, rolando fracture, thumb

## Abstract

Rolando fractures are comminuted, multifragmented, complete intra-articular fractures at the base of the thumb metacarpal. A review of the literature and our clinical practice shows that it can be treated in many ways. Patients with a non-displaced fracture require immobilization of the thumb. Nonoperative treatment is rare. Displaced fractures should be treated surgically. The primary goal of Rolando fracture treatment is to achieve anatomical reduction, ensuring that the fractured bones realign in their original anatomical position. As described in the article, the procedure of implanting an endoprosthesis into the carpometacarpal (CMC) joint of the thumb after an injury to the base of the first phalanx is an innovative method not yet described in medical sources. Our aim was to quickly return the patient to the full functional capacity of the upper limb while minimizing the risk of degenerative changes in the joint. We believe that although the proposed method is much more expensive compared to other surgical treatment techniques, it can be used in the treatment of young people, especially those with multifragmentary forms of fracture in whom there is a high risk of degenerative disease of the CMC joint.

## Introduction

In 1910, Italian surgeon Silvio Rolando was the first to describe a “Y-shaped” (Y-pattern) intra-articular fracture pattern of the first metacarpal base, which has since been named after him. Rolando fracture is referred to as a comminuted, multifragmented, complete intra-articular fracture at the base of the thumb metacarpal. The fracture line is Y or T-shaped. The second type of fracture in this area is Bennett fracture, which occurs on the ulnar side of the metacarpal base and is not multifragmented [1-5].

Analyzing the economic aspect, the availability of instruments and implants, the experience of orthopedic surgeons, and the frequency of complications, it is difficult to clearly indicate a method that would be inexpensive (paid), widely available, and not require extensive experience and manual skills of the surgeon. Certainly, the key effect of treatment should be patient satisfaction and improvement in the functional condition of the hand. Therefore, the primary goal of Rolando fracture treatment is to achieve anatomical reduction, ensuring that the fractured bones realign in their original anatomical position. Another important element of treatment is stable fixation, which allows for early mobilization, restoring the optimal range of motion and reducing pain [1,2].

Patients with a non-displaced fracture require only non-surgical treatment. This kind of treatment consists of immobilization with a brace or plaster cast of the thumb for a period of 4-6 weeks. Nonoperative treatment is rare. Displaced fractures should be treated surgically. This is due to the fact that this type of fracture is often accompanied by subluxations in the carpometacarpal (CMC) joint and additional damage to the ligamentous apparatus leading to degenerative changes. Various methods of surgical treatment are described in the literature. The oldest and cheapest treatment methods include closed reduction and immobilization with a brace or casting and closed reduction combined with percutaneous stabilization of Kirschner wires (K-wires). However, both methods do not guarantee full reconstruction of the articular surface. Other currently used surgical techniques include open reduction and internal fixation (ORIF) with a miniature T or L-plate, ligamentotaxis (ligamentous reduction of the fragments) with an external fixation or skeletal traction, combined use of external fixator with limited internal fixation and bone grafting, and arthroscopic-assisted technique for restoration of articular surface, which provides a greater chance of restoring the articular surface and reducing the risk of degenerative changes in the joint [1,3,5,6].

Accompanying the Rolando fracture, initial articular cartilage damage, adhesions, and contractures of soft tissues with progressive loss of joint space can lead to post-traumatic osteoarthritis and disability. However, there is no data on the incidence of degenerative changes after injuries in the area of the thumb CMC joint.

As described in the article, the procedure of implanting an endoprosthesis into the CMC joint of the thumb after an injury to the base of the first phalanx is an innovative method not yet described in medical sources. Our aim was to quickly return the patient to the full functional capacity of the upper limb while minimizing the risk of degenerative changes (osteoarthritis and disability) in the joint.

## Case presentation

**Figure 1:**
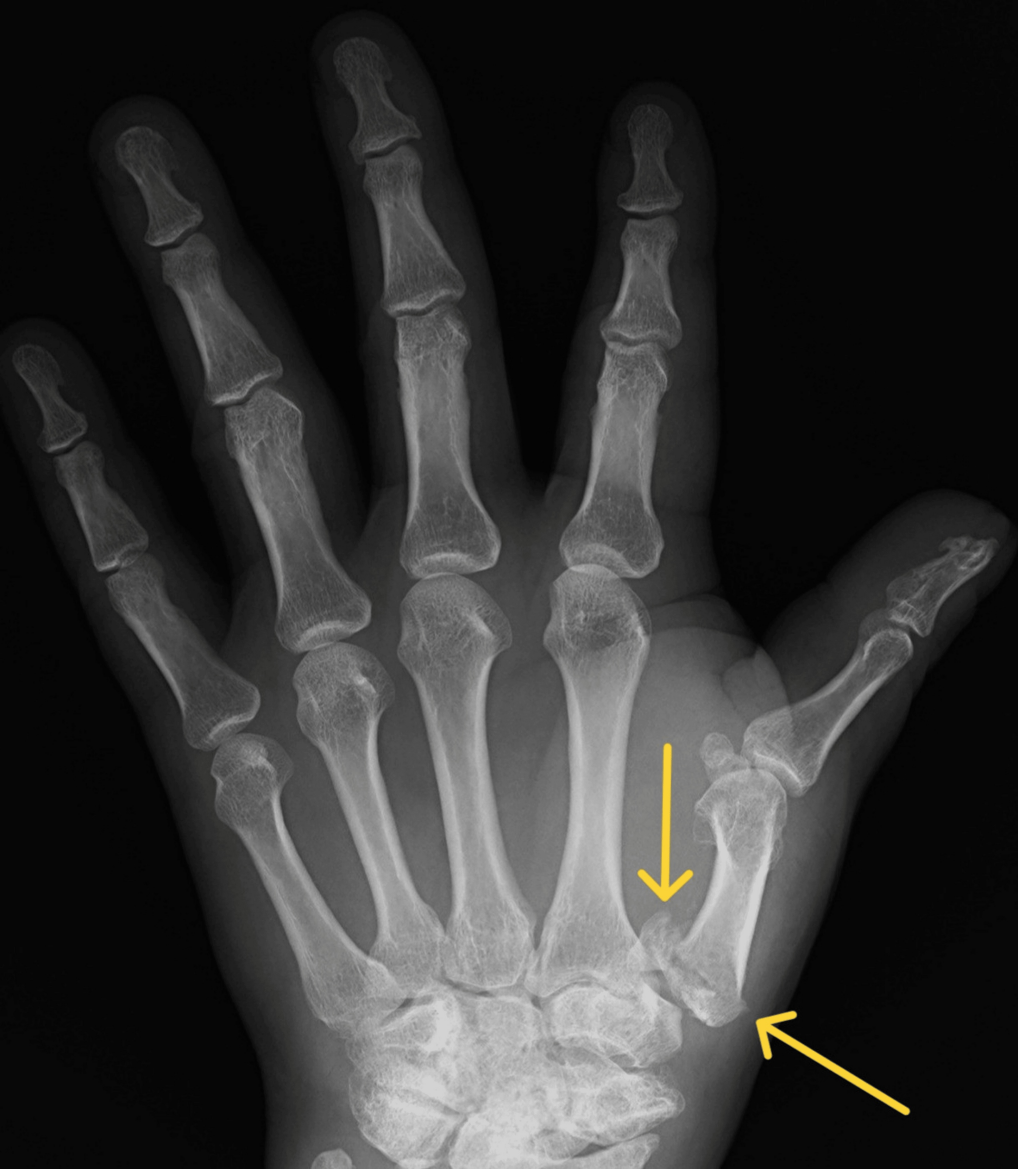
Rolando fracture with subluxation of the CMC joint of the first metacarpal bone (X-ray in A-P projection) CMC: carpometacarpal; A-P: anterior-posterior

**Figure 2:**
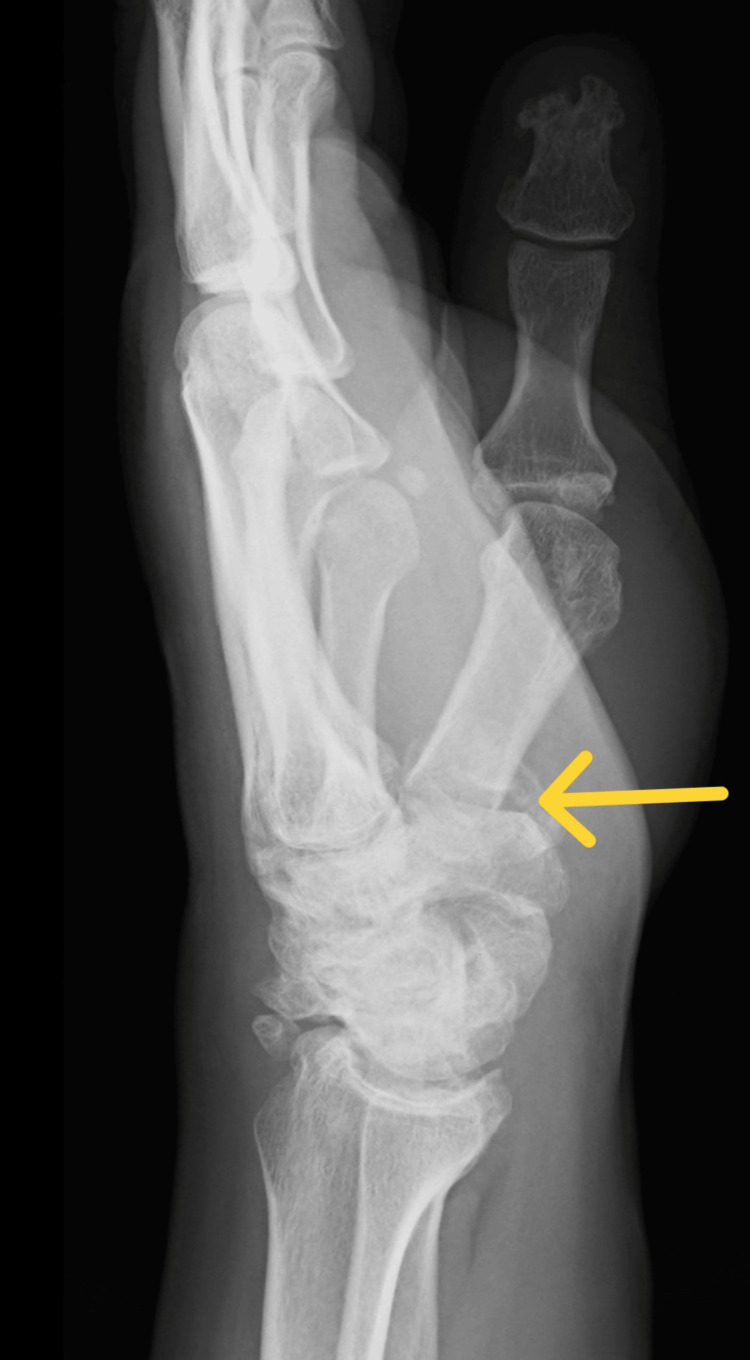
Rolando fracture with subluxation of the CMC joint of the first metacarpal bone (X-ray in lateral projection) CMC: carpometacarpal

The article describes the case of a 34-year-old patient hospitalized due to an extensive injury on the left thumb. The X-ray examination revealed a multifragmentary, trans-articular fracture of the proximal end of the first metacarpal bone, with subluxation of the CMC joint. Moreover, the study confirmed the presence of degenerative changes in the form of numerous osteophytes after a scaphoid fracture complicated by pseudarthrosis scaphoid non-union advanced collapse (SNAC III) (Figures 1-2).

**Figure 3:**
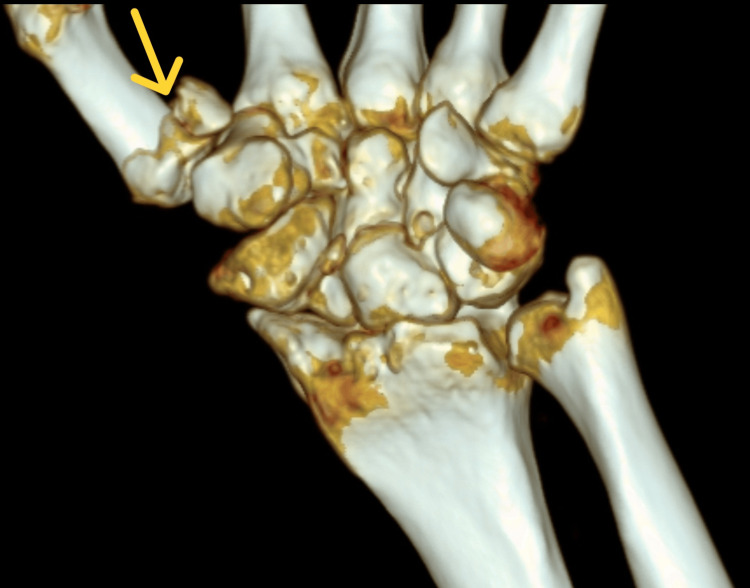
Reconstruction 3D based on computed tomography

**Figure 4:**
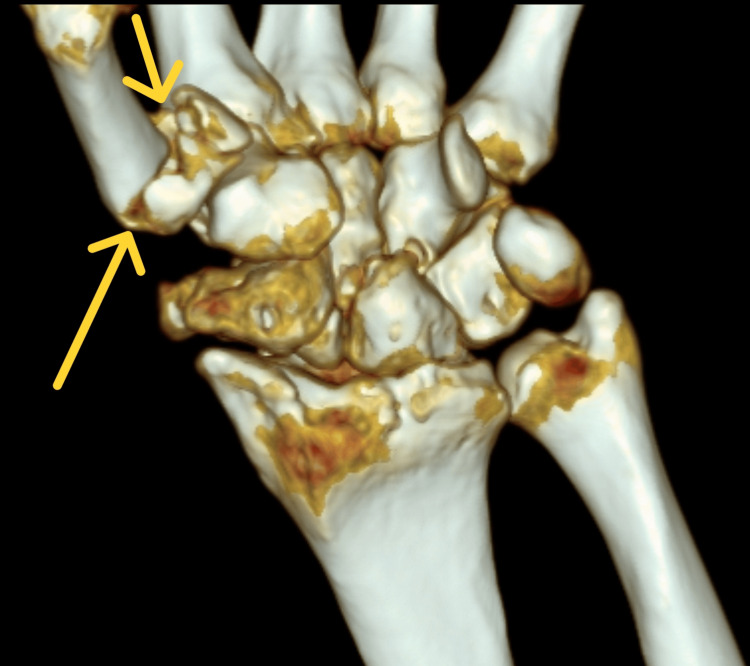
Reconstruction 3D based on computed tomography

In order to select the appropriate surgical treatment technique, the patient's diagnostics were extended to include computed tomography. On its basis, the course of the fracture line was precisely determined in 3D reconstruction (Figures 3-4).

**Figure 5:**
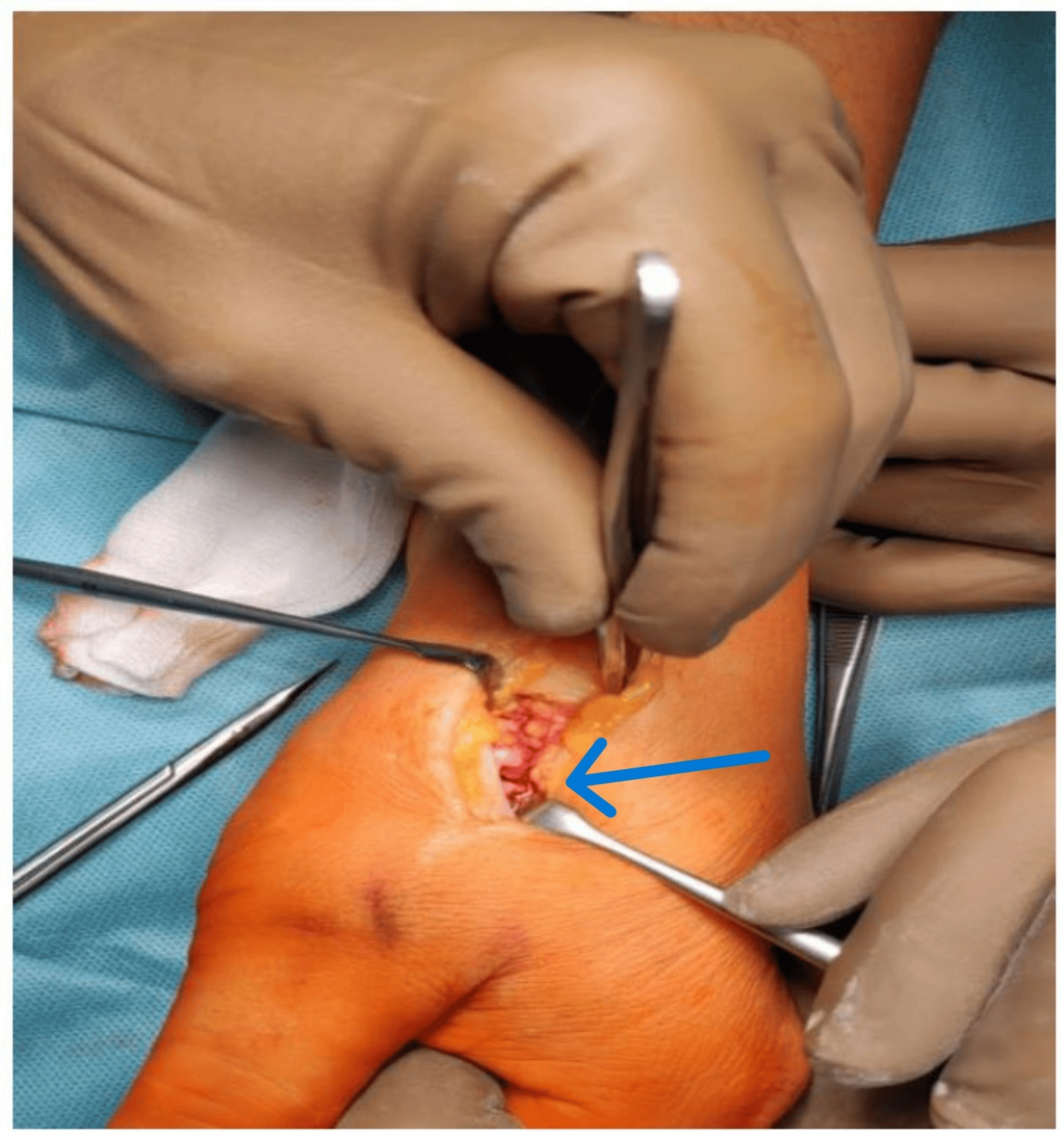
Preparation of soft tissues

In consultation with the patient, a decision was made to implant the dual mobility endoprosthesis into the CMC joint of the left thumb. Using measurements, the appropriate length and width of the implant were selected. Additionally, numerous osteophytes and interposing tissues were removed. The endoprosthesis was implanted using the press-fit method. The process of thorough preparation of soft tissues using a dorsal approach at the level of the CMC joint of the left thumb is depicted in Figure 5.

**Figure 6:**
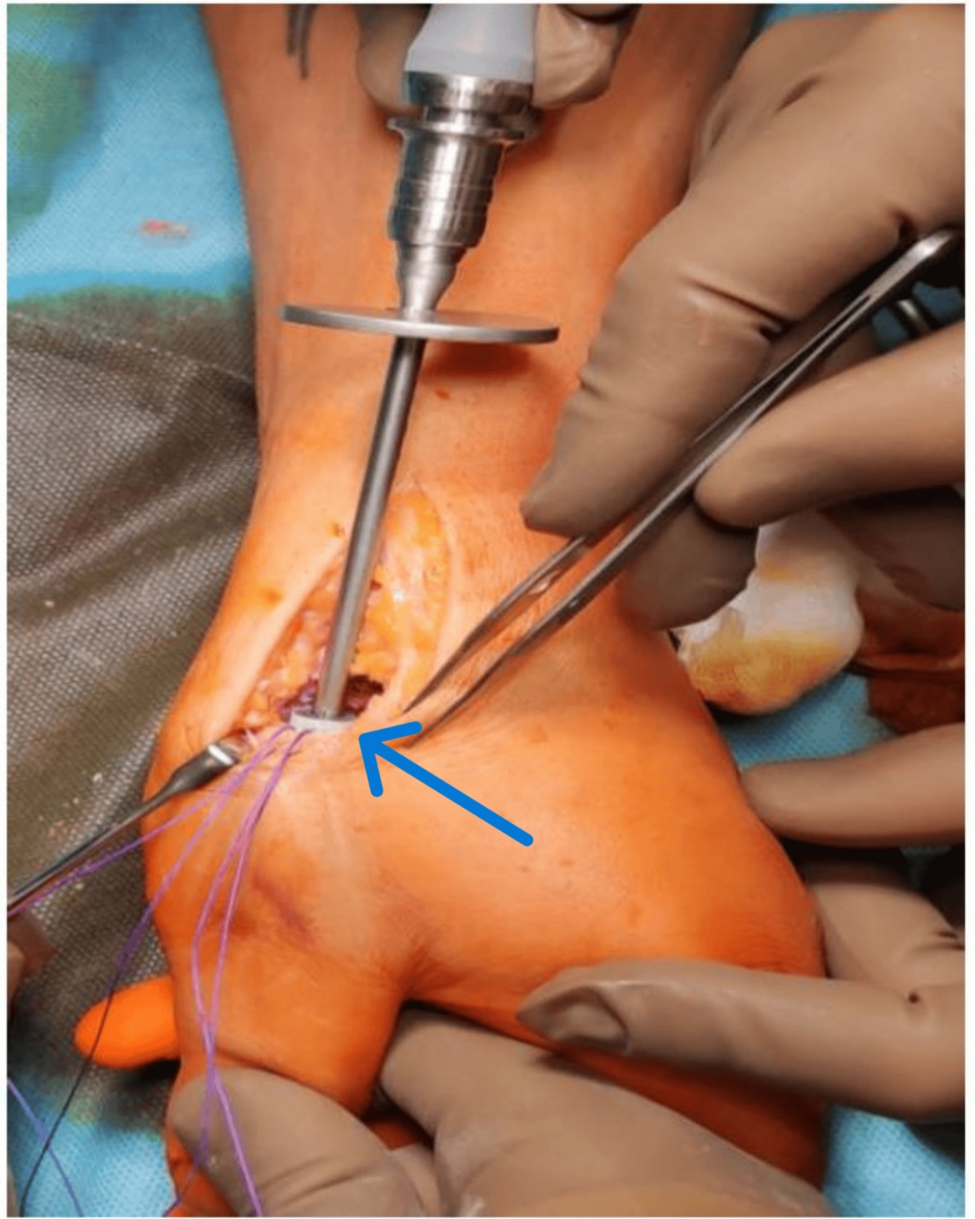
Implantation pin prosthesis into the first metacarpal bone

The next stage of the procedure was the implantation of the CMC thumb prosthesis pin into the first metacarpal bone using the press-fit method (Figure 6).

**Figure 7:**
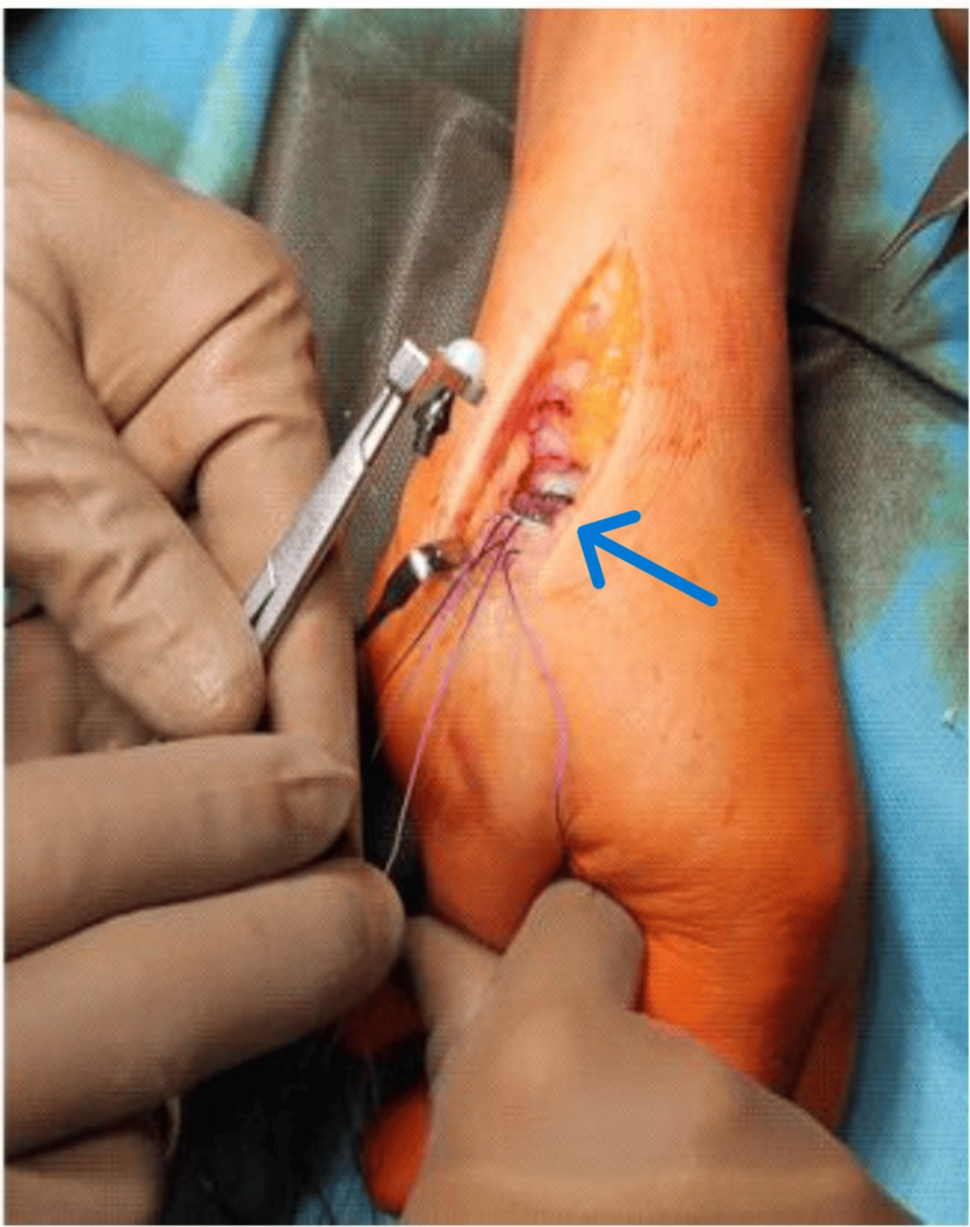
Implantation of the head of the prosthesis

The implantation of the head of a (double mobile) CMC thumb prosthesis is shown in Figure 7.

**Figure 8:**
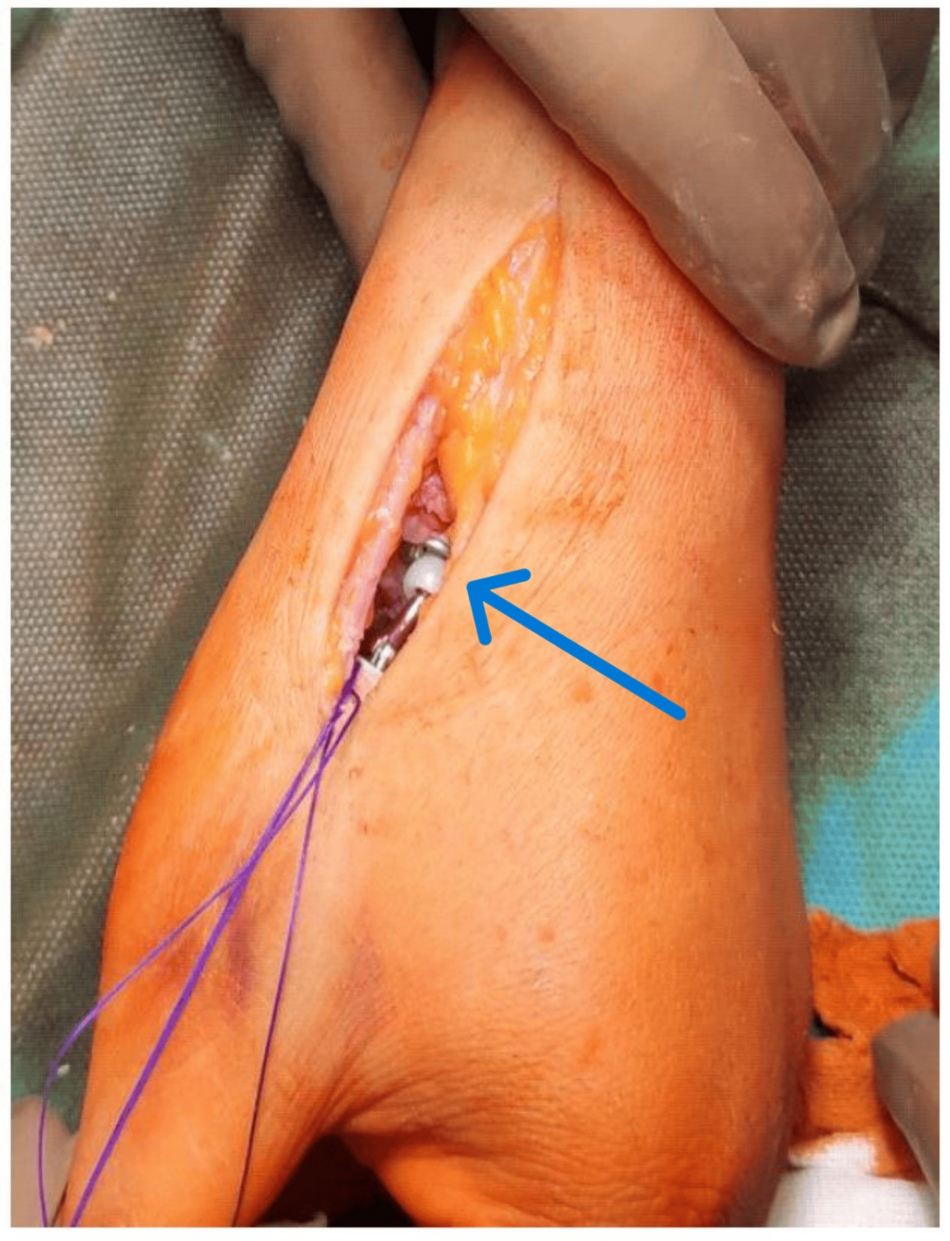
Repositioning for the final acceptance of the congruence of the prosthesis

Finally, the repositioning for the final acceptance of the congruence of the prosthesis elements was done (Figure 8).

**Figure 9:**
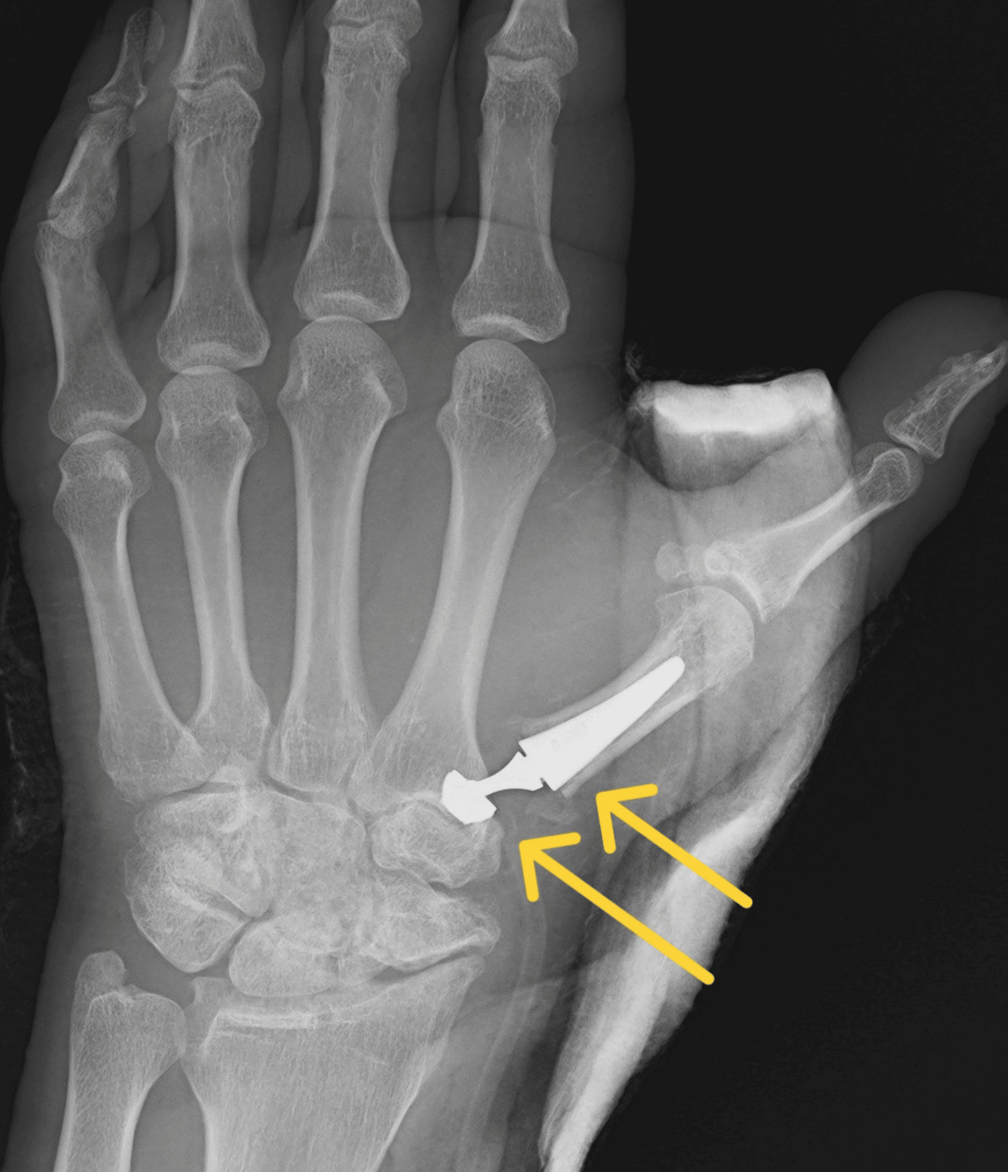
Control X-ray in A-P projection obtained after surgery A-P: anterior-posterior

**Figure 10:**
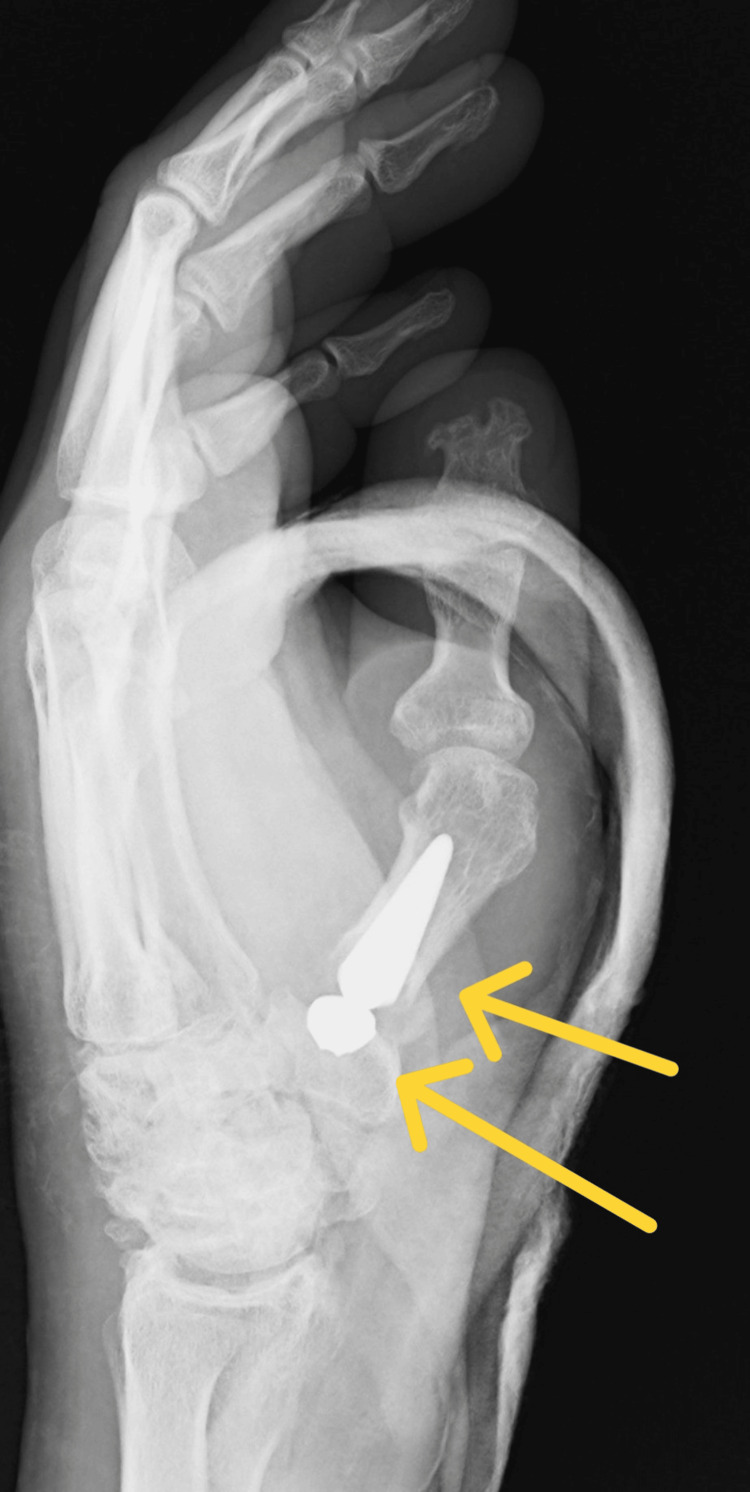
Control X-ray in lateral projection obtained after surgery

After the surgery, the patient's limb was immobilized in a special plaster splint for two weeks. Postoperative X-rays confirmed the correct positioning of the endoprosthesis elements (Figures 9-10).

After two weeks, the skin sutures and plaster splint were removed, allowing a full gradual range of motion of the thumb. At this stage of treatment, the patient was instructed on the principles of rehabilitation of the operated hand. At home, he performed the recommended active exercises twice a day. Additionally, he was directed to physical therapy treatments: low-frequency pulsed magnetic field and laser biostimulation. The therapeutic series included 10 procedures performed on the CMC joint area of the thumb.

In the fourth week of observation: intensity of rest pain on the numeric rating scale (NRS) was 3, functional pain in NRS was 5, range of motion in the sagittal and frontal planes was 35 degrees, oppositional range of motion was 5/10 according to the Kapanji score (a tool based on where the patient on their hand is able to touch with the tip of their thumb), global grip strength was 40 kg, and functional efficiency according to disabilities of the arm, shoulder, and hand (DASH) was 6.2/100.

After eight weeks, improvement was observed in all observed parameters. Long-term results (after 12 and 24 months) showed further improvement in the intensity of functional pain and the range of motion of the thumb opposition. The remaining assessed parameters obtained the same results as in previous observations. The lower value of global grip strength in the fractured limb may result from the fact that it was a non-dominant limb. The literature review showed that grip strength in the non-dominant hand may be up to 30% lower than in the dominant. Detailed results obtained by the patient are presented in Table 1.

**Table 1:** Results of the assessed parameters in subsequent observation periods NRS: numeric rating scale; DASH: disabilities of the arm, shoulder and hand

Parameter	4 weeks	8 weeks	12 months	24 months
NRS rest	3	1	1	1
NRS functional	5	3	0	0
Range of motion in the sagittal plane	35	45	45	45
Range of motion in the frontal plane	35	40	40	40
Opposition of the thumb according to Kapanji	5(10)	9(10)	10(10)	10(10)
Global grip strength, broken limb	40 kg	50 kg	48 kg	51 kg
Global grip strength, healthy limb	52 kg	51 kg	55 kg	54 kg
DASH	6.2(100)	4.5(100)	4.5(100)	4.5(100)

**Figure 11:**
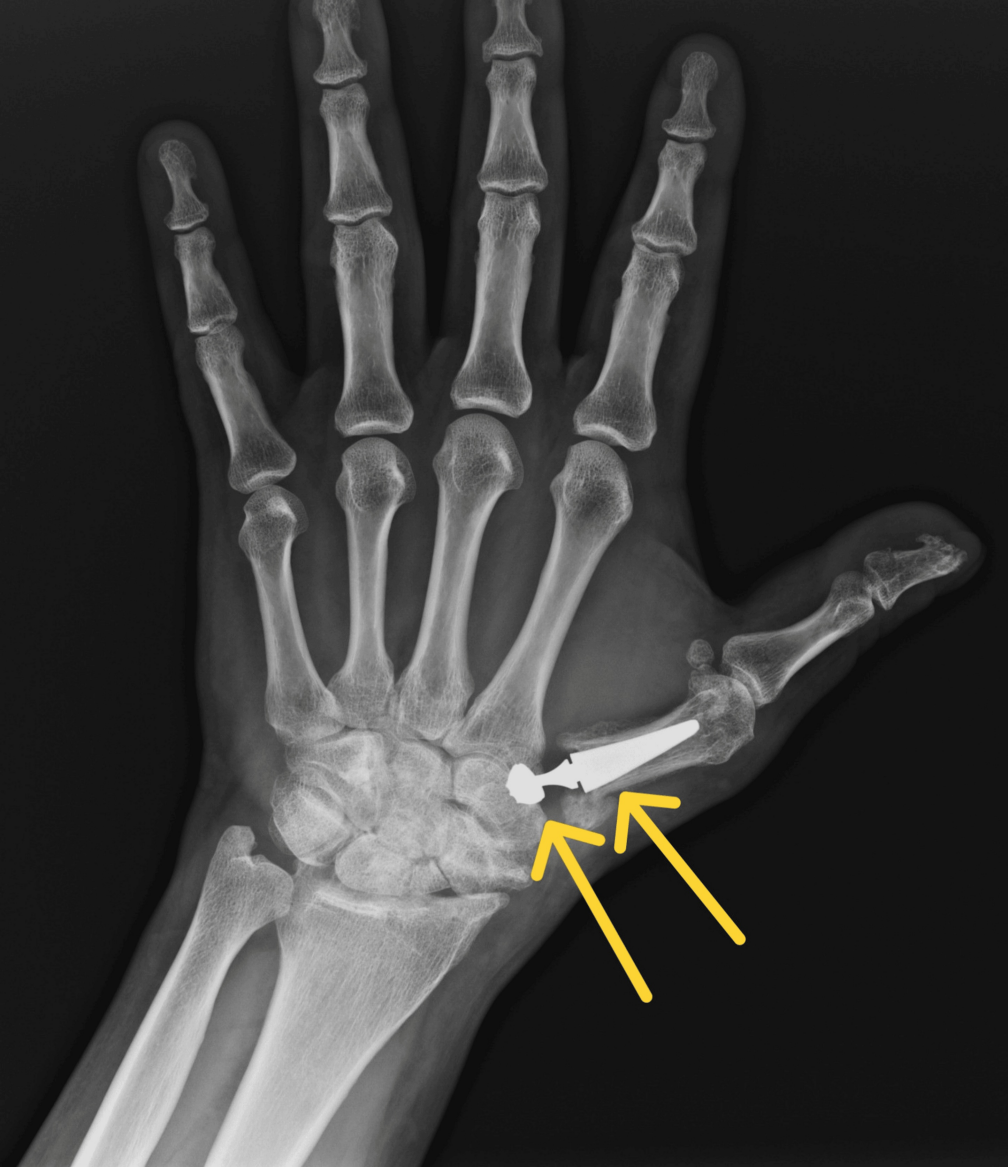
X-ray in A-P projection obtained two years after surgery A-P: anterior-posterior

**Figure 12:**
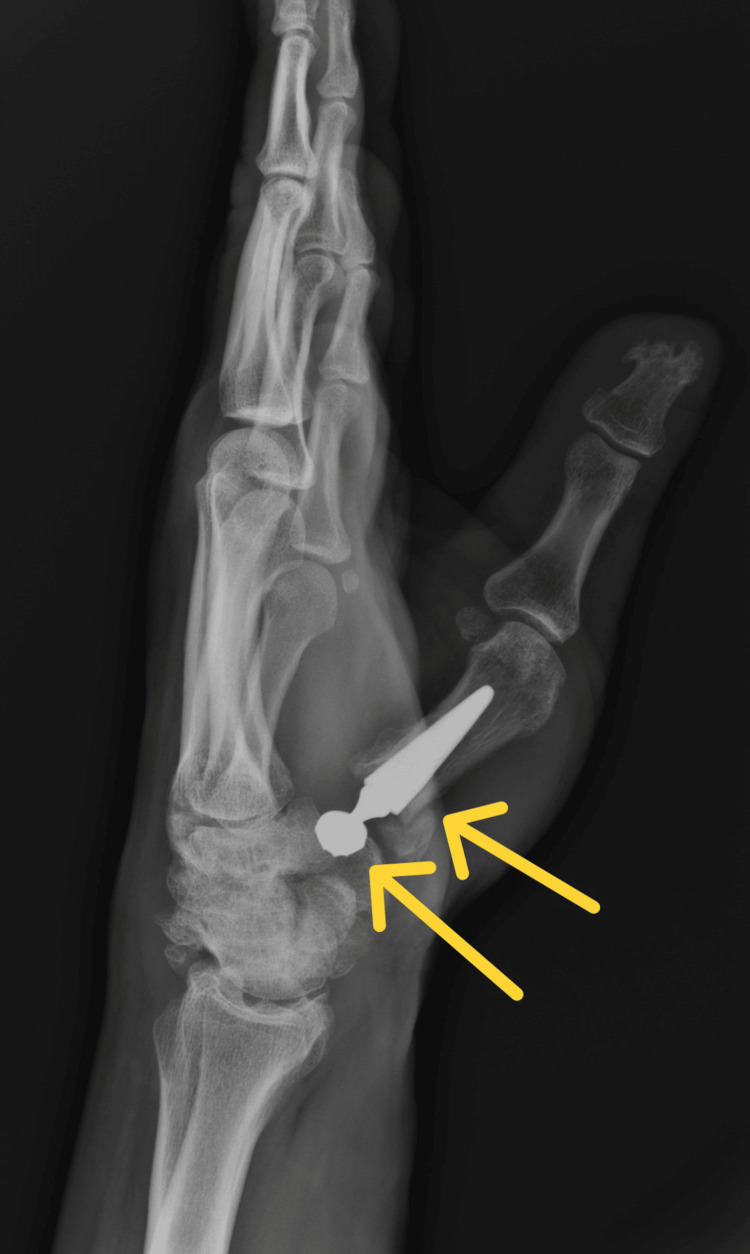
X-ray in lateral projection obtained two years after surgery

Control radiographic results obtained two years after surgery showed the correct positioning of the endoprosthesis, without any signs of instability (Figures 11-12).

## Discussion

Rolando fracture treatment depends on the severity of the thumb base crushing (crushing rate of the thumb’s base) and the degree of displacement of the crack. All kinds of treatments have advantages and disadvantages. It is not always possible to recreate the articular surface in the case of comminution of the fracture [6]. The main long-term consequences of a Rolando fracture include stiffness and osteoarthritis. This applies especially to multifragmentary forms of fracture [5]. This was confirmed by a literature review conducted by Van Niekerk et al. who wrote that some authors have described severe posttraumatic osteoarthritis and disability in patients with more than 1 mm of residual articular surface incongruity [7]. In 1991, Langhoff et al. reported results on 17 Rolando fractures. Restoration of the articular surface was the goal of open treatment when closed reduction was inadequate. An open technique was required in 11 of 14 (79%) patients. Long-term radiographic follow-up (after six years) performed on 11 patients showed that six (54%) had evidence of arthritic changes [8]. Mumtaz et al. reported on nine patients who underwent ORIF using a miniplate. The functional outcomes were good in eight. In one patient, the effect of the surgery was unsatisfactory due to limited range of motion of the thumb and pain. In a follow-up examination, the patient was diagnosed with stage 3 Eaton-Littler degenerative disease. The authors suggested that the ORIF technique with miniplate offers many benefits. The most important includes accurate anatomical reconstruction of the articular surface. But its use is not advisable in every case. It is especially recommended for multifragmentary forms of fracture. The use of this method allows for early mobilization of the hand, thus minimizing the risk of complications such as stiffness and degenerative joint disease. The authors emphasized, however, that the described technique requires high precision [1].

Referring to the data presented above, we believe that although the proposed method is much more expensive compared to other surgical treatment techniques, it can be used in the treatment of young people, especially those with multifragmentary forms of fracture in whom there is a high risk of degenerative disease of the CMC joint. In addition, it is worth mentioning that a Rolando fracture may result in significant progression of osteoarthritis of the joint, which may result in the need for another surgery in the future.

## Conclusions

Implantation of the CMC endoprosthesis in the patient with Rolando fracture reduced the intensity of pain in the wrist and thumb area, restored the normal range of joint mobility, and improved the efficiency of the upper limb.
